# Models of Chemical Communication for Micro/Nanoparticles

**DOI:** 10.1021/acs.accounts.3c00619

**Published:** 2024-03-01

**Authors:** Jordi Ventura, Antoni Llopis-Lorente, Loai K. E. A. Abdelmohsen, Jan C. M. van Hest, Ramón Martínez-Máñez

**Affiliations:** † Instituto Interuniversitario de Investigación de Reconocimiento Molecular y Desarrollo Tecnológico (IDM), Universitat Politècnica de València, Universitat de València, Camino de Vera s/n, 46022 València, Spain; ‡ Unidad Mixta de Investigación en Nanomedicina y Sensores, Universitat Politècnica de València, Instituto de Investigación Sanitaria La Fe, Av Fernando Abril Martorell 106, 46026 Valencia, Spain; § Department of Chemical Engineering & Chemistry, Department of Biomedical Engineering, Institute for Complex Molecular Systems (ICMS), Eindhoven University of Technology, Het Kranenveld 14, 5600 MB Eindhoven, The Netherlands; ∥ Unidad Mixta UPV-CIPF de Investigación en Mecanismos de Enfermedades y Nanomedicina, Universitat Politècnica de València, Centro de Investigación Príncipe Felipe, C/Eduardo Primo Yúfera 3, 46100 Valencia, Spain; ⊥ CIBER de Bioingeniería, Biomateriales y Nanomedicina (CIBER-BBN), Instituto de Salud Carlos III, 28029 Madrid, Spain

## Abstract

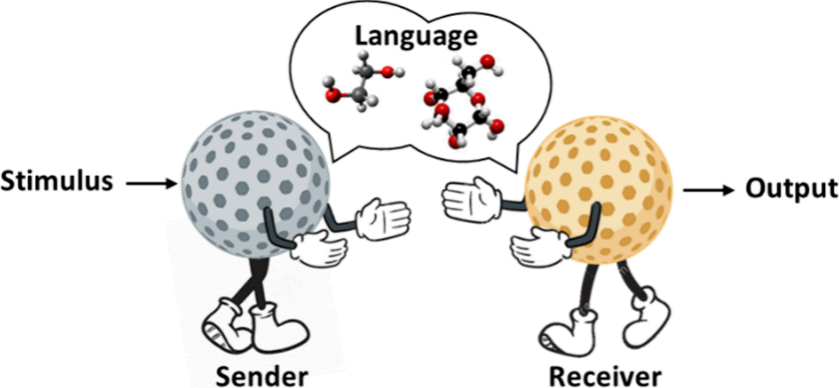

Engineering chemical communication between micro/nanosystems (via the exchange of chemical messengers) is receiving increasing attention from the scientific community. Although a number of micro- and nanodevices (e.g., drug carriers, sensors, and artificial cells) have been developed in the last decades, engineering communication at the micro/nanoscale is a recent emergent topic. In fact, most of the studies in this research area have been published within the last 10 years. Inspired by nature—where information is exchanged by means of molecules—the development of chemical communication strategies holds wide implications as it may provide breakthroughs in many areas including nanotechnology, artificial cell research, biomedicine, biotechnology, and ICT. Published examples rely on nanotechnology and synthetic biology for the creation of micro- and nanodevices that can communicate. Communication enables the construction of new complex systems capable of performing advanced coordinated tasks that go beyond those carried out by individual entities. In addition, the possibility to communicate between synthetic and living systems can further advance our understanding of biochemical processes and provide completely new tailored therapeutic and diagnostic strategies, ways to tune cellular behavior, and new biotechnological tools. In this Account, we summarize advances by our laboratories (and others) in the engineering of chemical communication of micro- and nanoparticles. This Account is structured to provide researchers from different fields with general strategies and common ground for the rational design of future communication networks at the micro/nanoscale. First, we cover the basis of and describe enabling technologies to engineer particles with communication capabilities. Next, we rationalize general models of chemical communication. These models vary from simple linear communication (transmission of information between two points) to more complex pathways such as interactive communication and multicomponent communication (involving several entities). Using illustrative experimental designs, we demonstrate the realization of these models which involve communication not only between engineered micro/nanoparticles but also between particles and living systems. Finally, we discuss the current state of the topic and the future challenges to be addressed.

## Key References

- Gimenez, C.; Climent, E.; Aznar, E.; Martínez-Máñez, R.; Sancenon, F.; Marcos, M. D.; Amorós, P.; Rurack, K. Toward chemical communication between gated nanoparticles. *Angew. Chem., Int. Ed.***2014**, *53*, 12629–12633.^1^ A cascade communication model was developed using mesoporous silica nanoparticles that only release their cargo in the presence of a specific stimulus.
- de Luis, B.; Llopis-Lorente, A.; Rincón, P.; Gadea, J.; Sancenón, F.; Aznar, E.; Villalonga, R.; Martínez-Máñez, R. An interactive model of communication between abiotic nanodevices and microorganisms. *Angew. Chem., Int. Ed.***2019**, *58*, 14986–14990.^2^ An interactive communication system was developed in which yeast cells exchange information with an enzyme-controlled nanodevice and express GFP in response.
- Buddingh’, B. C.; Elzinga, J.; van Hest, J. C. M. Intercellular communication between artificial cells by allosteric amplification of a molecular signal. *Nat. Commun.***2020**, *11*, 1652.^3^ Long distance communication between two artificial cell populations was enabled, using the AMP molecules produced by senders as an allosteric activator in receivers.
- Estepa-Fernández, A.; García-Fernández, A.; Lérida-Viso, A.; Morellá-Aucejo, Á.; Esteve-Moreno, J. J.; Blandez, J. F.; Alfonso, M.; Candela-Noguera, V.; Vivo-Llorca, G.; Sancenón, F.; Orzáez, M.; Martínez-Máñez, R. Engineering nanoparticle communication in living systems by stigmergy: An application to enhance antitumor therapy in triple-negative breast cancer. *Nano Today***2023**, *48*, 101692.^4^ We designed two populations of nanoparticles that communicate indirectly through the modification of the environment *in vivo*, to achieve an enhanced therapeutic effect in breast cancer cells.

## 1 Introduction

The engineering of chemical communication between micro/nanosystems addresses a key question at the forefront of research in molecular sciences: how to evolve toward the next generation of advanced micro/nanoparticles capable of exchanging information with other micro/nanoparticles or with living cells. Over the last two decades, the design of micro/nanoparticles has drawn attention due to their potential applications in diverse areas such as biomedicine, sensing, and antimicrobials. In addition, engineering communication capabilities offers the potential to enable cooperation between micro/nanoparticles, collective actuation, and constructing programmable multicomponent systems.^5^ In the context of synthetic biology, engineering communication is a key aspect for the development of artificial cells (responsive compartmentalized systems containing biological or synthetic machinery) that mimic the functionality and dynamicity of living cells. In fact, the topic of chemical communication has recently attracted attention from artificial cell researchers and has been discussed in several mini-review/opinion-type articles.^6−11^ Furthermore, micro/nanoparticles able to communicate with living cells could be employed to tune cellular behavior, sense changes in cellular states, and design a new generation of therapeutic delivery systems.

Chemical communication, based on the exchange of information via the use of molecules, is the main way of communication for living systems.^12^ For instance, bacteria communicate with peers from the same species by secreting specific quorum sensing molecules that regulate population behavior. In addition, the functioning of multicellular organisms relies on a wide range of cellular communication processes. These natural chemical messengers are diverse, ranging from neurotransmitters (such as acetylcholine and dopamine) released by neurons to communicate with neighbors and with muscle cells to hormones (such as insulin and adrenaline) secreted by regulatory cells to activate certain processes in distant receiver cells. Thus, cellular communication processes offer inspiration for engineering chemical communication between micro/nanoparticles and between micro/nanoparticles and cells.

**Figure 1:**
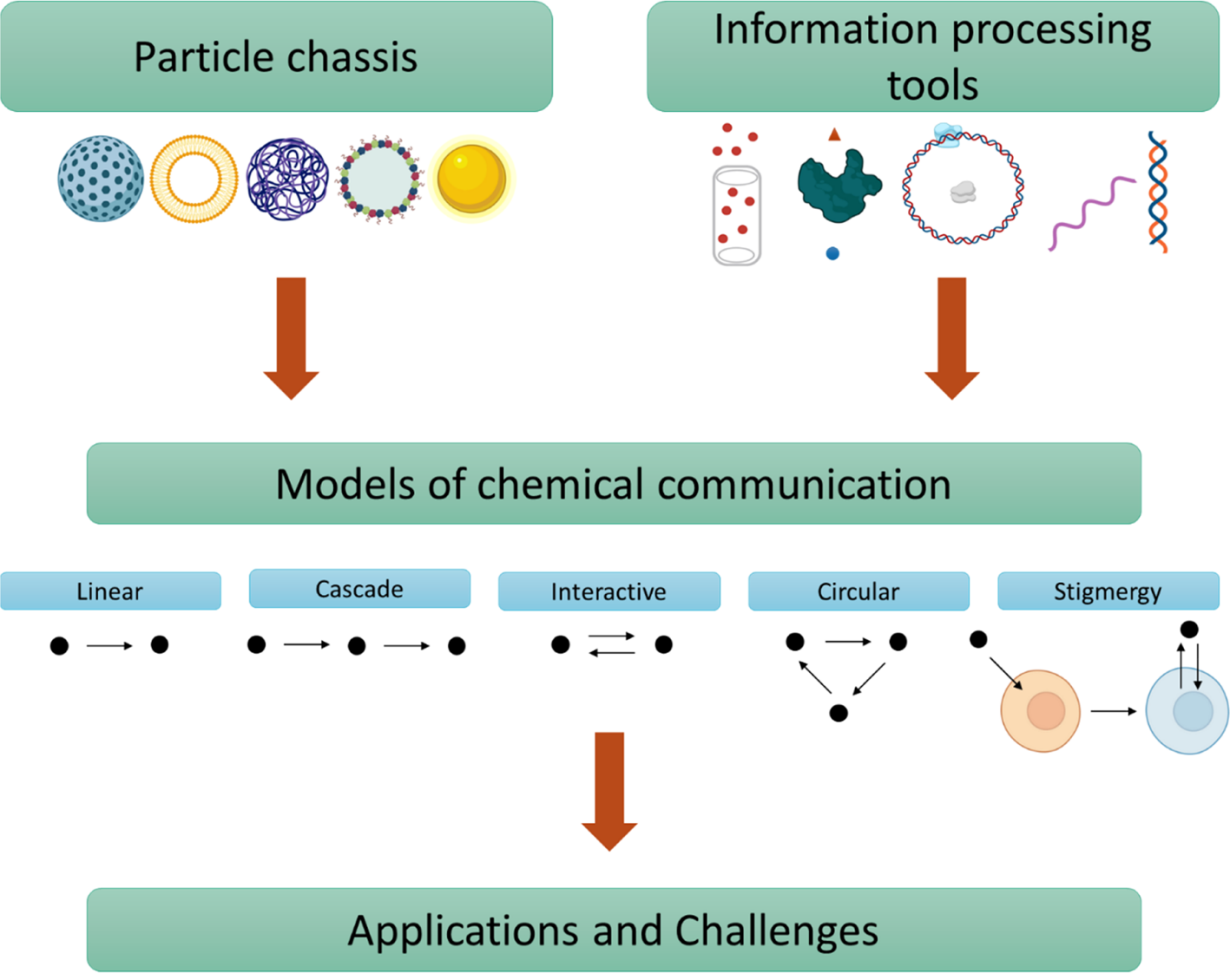
Outline and flow for engineering chemical communication with micro/nanoparticles.

This Account covers recent advances in the design of various models of communication for micro/nanoparticles (Figure 1). Herein, we describe and classify chemical communication systems involving micro/nanoparticles based on the pathway followed by chemical messengers—a different approach to previous reviews in this area.^5,13,14^ The works presented here are mainly from our own (based on the use of silica nanoparticles, coacervates, and lipid vesicles) but also include selected examples by others using different types of particles (e.g., proteinosomes by de Greef and Mann groups) and molecular machinery (e.g., protein synthesis machinery by Mansy’s group). We start by briefly summarizing the utility of different materials and molecular machinery to engineer particles with communication capabilities. We propose different main models of communication based on the directional flow of chemical messengers (linear, cascade, interactive, or circular) and communication by stigmergy and discuss reported examples in each category. These advances suggest that it would soon be possible to have a toolbox of strategies to design communication systems based on tailor-made micro/nanoparticles for specific applications. Moreover, in the final section, we discuss challenges to be addressed in the engineering of chemical communication systems.

## 2 Tools to Engineer Chemical Communication

Micro/nanoparticles can be designed to have different actuation mechanisms in communication networks by functioning as (i) smart delivery systems, able to release an entrapped chemical messenger, a dye, or a drug in response to a trigger; (ii) semipermeable compartments, able to entrap macromolecules such as enzymes while allowing the diffusion of small messengers (enzymatic products); and (iii) micro/nanoscaffolds to anchor responsive ensembles such as DNA strands or enzymes. As we discuss below in this section, the particle chassis and information processing tools (molecular components to process external information) are two main factors that correlate with the particle’s actuation mechanism in the communication network.

### 2.1 Particle Chassis

**Figure 2:**
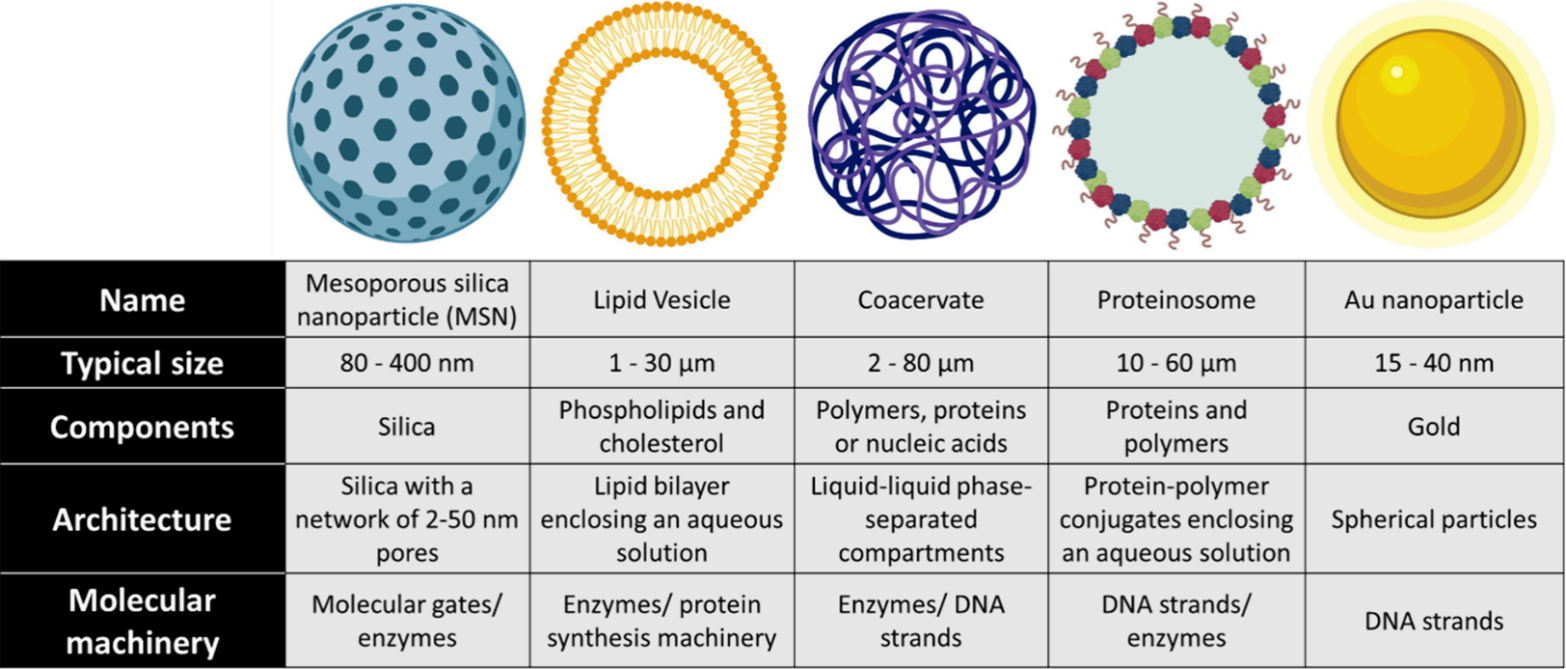
Different types of particle-chassis used to prepare micro/nanoparticles suitable for chemical communication (drawings are not to scale).

Different types of micro- and nanoparticles have been employed to construct chemical communication ensembles. They are based both on inorganic materials, such as silica or Au nanoparticles, and organic compartments, such as lipid vesicles, coacervates, and proteinosomes (Figure 2). Although here we use the term micro/nanoparticles in general, it should be noted that the term artificial (or synthetic) cells is often used to refer to organic micrometer-sized compartments with a certain degree of complexity and cell-like features.

**Mesoporous silica nanoparticles** (MSNs) have a typical diameter of around 100 nm and a pore size of around 2–3 nm; yet particles with a larger diameter (e.g., 400 nm) and pores (up to 50 nm) can be prepared if needed. They offer several advantages such as their high loading capacity (with chemical messengers, dyes or drugs), thermal stability, biocompatibility, and possibility of functionalization with gating ensembles (see section 2.2), making them highly appealing for developing chemical communication systems as shown by Martínez-Máñez’s group.^15^ Furthermore, MSNs can also be coupled with other nanoparticles like Au or platinum to develop more sophisticated Janus-type nanocarriers (with two different surfaces) capable of incorporating enzymes and working as nanomotors.^16,17^ Yet, MSNs cannot encapsulate in their pores high molecular mass compounds such as large proteins or long DNA structures due to the small pore size.

In turn, **lipid vesicles** (also known as liposomes) are organic compartments made of a lipid bilayer enclosing an aqueous solution (containing the desired cargo).^18^ Whereas nanoscale lipid vesicles have been widely used as nanocarriers, giant unilamellar vesicles (GUVs) are microvesicles recognized as one of the most promising platforms for assembling artificial cells. GUVs have a spatial organization (lipid membrane and aqueous lumen) and size (1–20 μm) comparable to natural cells.^19^ Their cell-like size allows visualization using optical microscope techniques to investigate chemical communication processes.^20^ Their interior can allocate macromolecules and small cargos to perform a wide range of reactions. However, GUVs are not suitable for encapsulating nonpolar small molecules which permeate through lipid membranes. Besides, their lower stability compared to that of MSNs may limit their use under harsh conditions.

**Coacervate** microdroplets are liquid–liquid phase-separated compartments made by the self-assembly of oppositely charged polyelectrolytes. Coacervates are also a promising artificial cell platform due to their ability to sequester macromolecules (such as proteins and DNA) in their highly crowded interior,^21^ while small molecules can diffuse inside and be released, which makes them suitable for gene expression and enzymatic reactions.^22,23^ Their main disadvantage is their limited stability over time, under high ionic strength and upon pH changes which affect the interactions between their components. In this area, van Hest’s group pioneered the development of membranized amylose-based coacervate microdroplets stabilized with a synthetic triblock copolymer that have been used in several examples of communication.^21,23^

Although our groups have mainly focused on the utility of MSNs, GUVs and coacervates in the reported examples of chemical communication; other materials also hold potential in this area. De Greef, Mann and colleagues have pioneered the deployment of protein-based microcapsules known as **proteinosomes**. In contrast to lipid vesicles, proteinosomes are permeable to short single-stranded DNA sequences, making them ideal for protocell DNA-based communication.^24,25^**Au nanoparticles** have been used as supports where molecules like DNA can be attached for chemical communication.^26,27^ They are highly stable, but their use in communication models has been restricted to the use of DNA strands. Moreover, other types of particles such as polymeric, metallic, and hybrid particles could be used in the design of chemical communication systems in the near future.

### 2.2 Molecular Machinery

**Figure 3:**
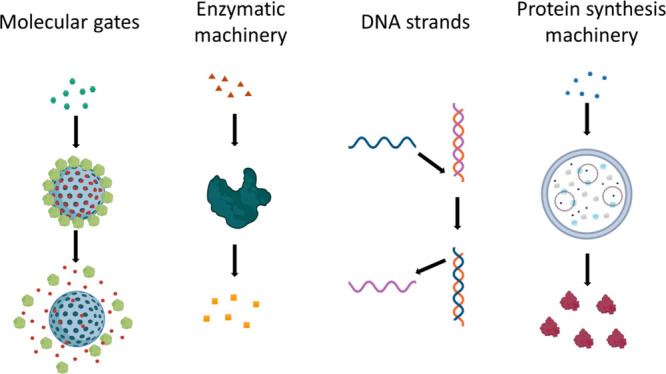
Information processing tools used in micro/nanoparticles suitable for chemical communication.

Micro/nanoparticles for chemical communication need to be able to read molecular inputs from the environment and produce a selective response. To do so, micro/nanoparticles should be equipped with molecular machinery of either synthetic or biological origin, enabling them to act as information processing tools (Figure 3).

Martínez-Máñez’s group pioneered the design of **molecular gates** as stimuli-responsive (supra)molecular ensembles that control the release of cargo from porous materials.^28,29^ These molecular gates (also known as gatekeepers, nanovalves, or gating ensembles) have been traditionally used as smart delivery systems in nanomedicine and sensing applications. Indeed, a rich variety of molecular gates that respond to physical (light, temperature, magnetic fields, etc.), chemical (pH changes, redox species, small molecules, etc.) and biochemical (enzymes, DNA, etc.) stimuli have been developed.^15^ Molecular gates hold great potential to engineer chemical communication between particles and with cells, as the entrapped cargo can act as a messenger for the next entity in the communication system.^1,30−32^

Chemical communication can also be achieved using micro/nanoparticles equipped with different **enzymatic machinery**. In this approach, enzymatic substrates and products act as chemical messengers in communication processes between particles—in this way, using an enzymatic reaction, a particle senses a stimulus and encodes a new message. For instance, different populations of coacervates equipped with glucose oxidase and peroxidase, respectively, were shown to be able to exchange H_2_O_2_ to produce a fluorescent product.^33^ A more complex enzymatic network was utilized to achieve allosteric communication between GUVs.^3^ Interestingly, enzymes can be attached to gated nanoparticles to control the opening of molecular gates with enzymatic products as implemented in some communication models (*vide infra*).^30,34,35^

Another strategy to enable different communication networks with high specificity and programmability is the use of **DNA strands** as messengers in displacement reactions.^24^ This strategy has been implemented in proteinosomes, coacervates and Au nanoparticles, where short single-stranded DNA sequences released by sender particles can hybridize and displace DNA sequences in receiver particles.^24,36,37^

Communication can also be engineered using a synthetic biology approach, based on the expression of proteins inside vesicles by means of transcription-translation (TXTL) reactions.^18,38^ In these reactions, **protein synthesis machinery** is encapsulated together with the DNA sequences encoding the proteins of interest. Upon rational design of the plasmid, protein expression can be activated in the presence of certain species (such as membrane-permeable quorum sensing molecules)^39^ or upon application of light.^40^ Then, the *in situ* expressed proteins can either form pores on the membrane to induce the release of entrapped cargo or catalyze certain reactions to produce chemical messengers.^41,42^

## 3 Models of Chemical Communication

**Figure 4:**
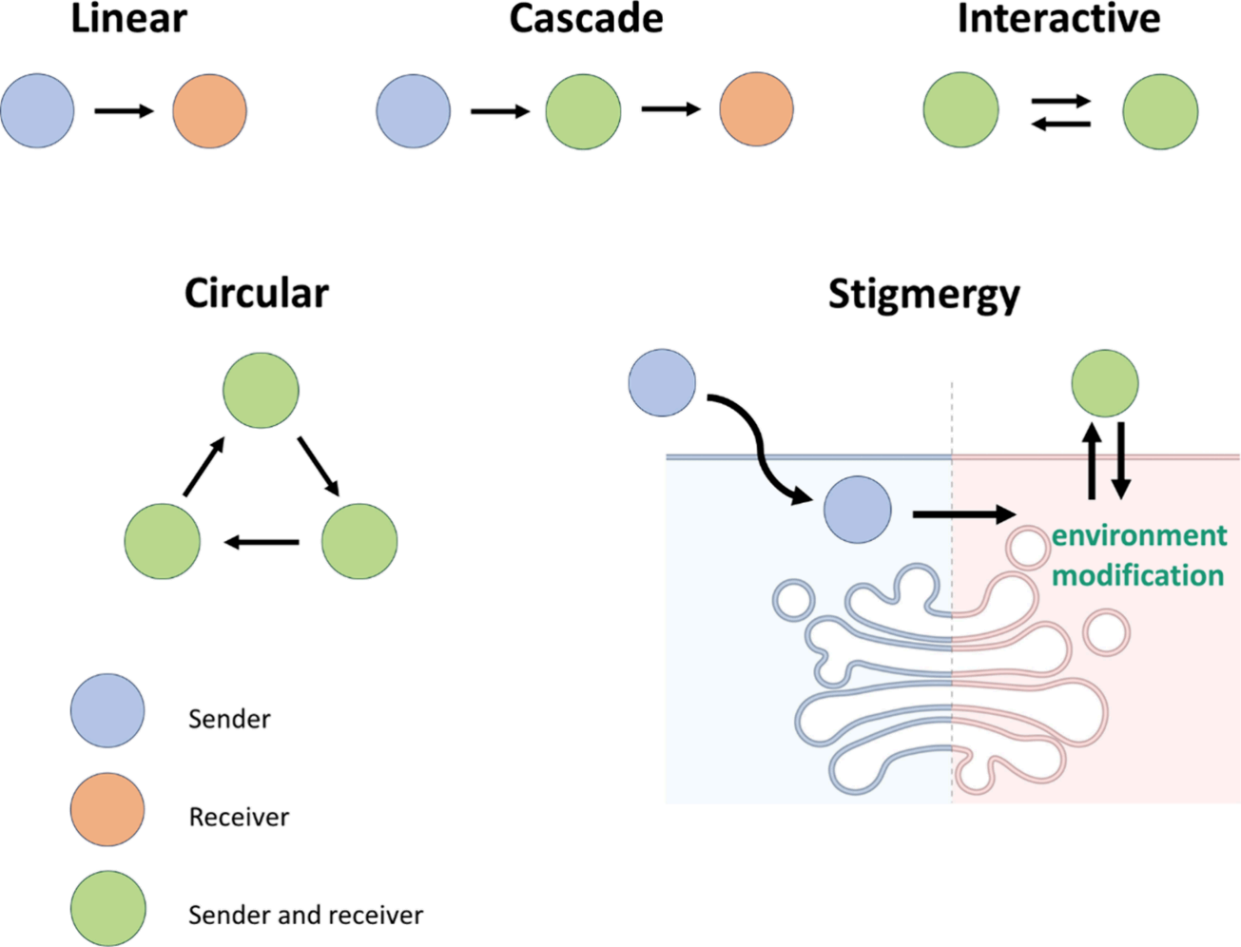
Models of chemical communication for micro/nanoparticles and cells. The circles represent the cells and particles involved in each model. In stigmergy, the environment modification refers to a change in the interior of an eukaryotic cell.

As exemplified by the contributions from our groups, we propose a classification of micro/nanoparticles’ communication based on the pathway followed by molecular information. This classification results in 5 main models (Figure 4): linear, cascade, interactive, circular and stigmergic. As the simplest, **linear** communication refers to a one-way flow of information from a sender to a receiver that responds to the perceived chemical message by producing a certain action (e.g., release of a cargo), but there is no response sent back to the sender. Based on concatenated linear communication events, the **cascade** model involves at least three entities (or potentially more) that communicate in a sequential fashion (i.e., particle 1 to particle 2 and then particle 2 with particle 3); thereby, cascade communication involves particles with a double receiver–sender role able to recognize a messenger from the previous particle/cell in the network and subsequently produce a message for the next particle/cell. In contrast, the **interactive** model involves a bidirectional flow of information where the sender channels a message to the receiver, and subsequently, the receiver decodes the message and produces a response that diffuses back to the original sender. When this message is capable of not only inducing a response in the original sender but also modifying its functioning, we refer to this process as feedback. In the **circular** model, the final output is produced by the first particle of the network after a hierarchical flow of information involving at least three particles (or potentially more). Interestingly, the circular flow of information between particles is reminiscent to biochemical cycles; however, experimental examples still lack the regulation and recycling observed in natural processes. Finally, for the **stigmergy** approach, we took inspiration from communication in natural swarm systems such as ants or bees. Stigmergy differs from previous models (where communication takes place via the direct exchange of messengers), since it does not involve direct interaction between the communicating entities. In contrast, in stigmergy, the first agent (nanoparticle) leaves a trace and induces changes in the medium (for instance, in a cell) that stimulate the action of the second agent (nanoparticle). In this section, we present illustrative examples in which micro/nanoparticles communicate following these different models of communication.

### 3.1 Linear Communication

**Figure 5:**
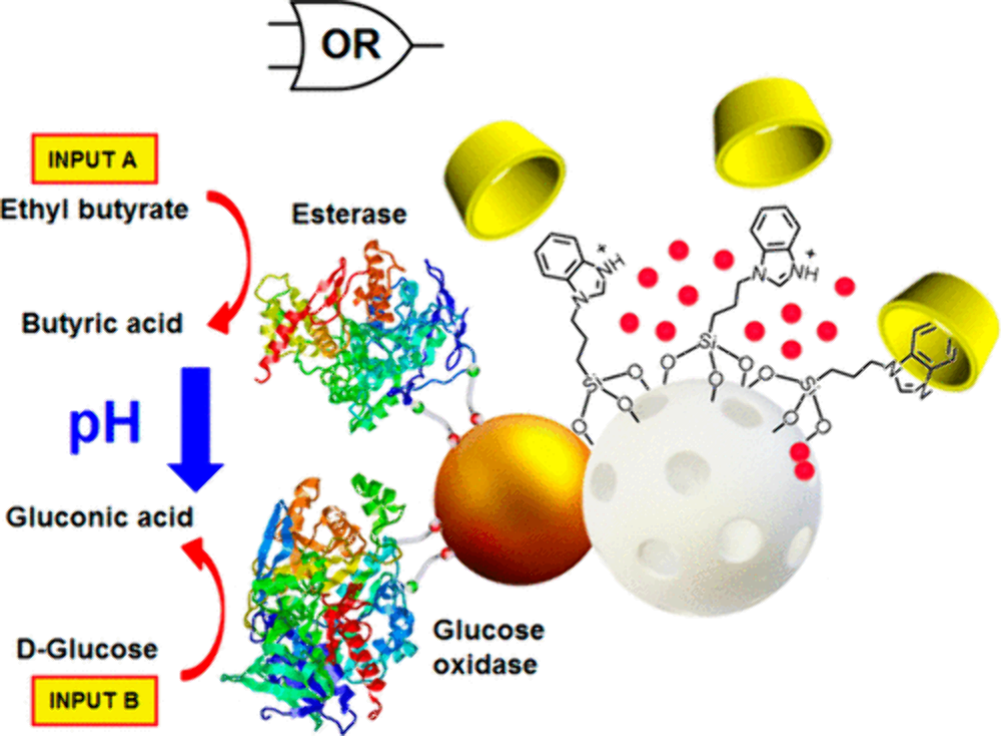
Schematic representation of communication on Janus Au-MSNs, where enzymatic effectors (glucose oxidase and esterase) on the Au surface sense glucose or ethyl butyrate as inputs, producing a message to open the pH-sensitive molecular gate on the MSN face. Reprinted with permission from ref (43). Copyright 2014 American Chemical Society.

In a collaboration between Villalonga and Martínez-Máñez’s group in 2014, a simple example of linear communication between two nanoparticles (Au and MSNs) that were chemically attached (Janus Au-MSNs) was reported. In this example, **enzymatic units** on the Au nanoparticle produced a chemical messenger that triggered the opening of the **molecular gate** on the silica nanoparticle (Figure 5).^43^ The Au unit acts as a control unit by reading information on the environment (enzymatic substrates) and transforming it into new chemicals that induce cargo release from the nanocarrier. In this example, esterase and glucose oxidase enzymes were immobilized on the Au surface, whereas the MSN surface was functionalized with benzimidazole groups and capped via the formation of an inclusion complex with β-cyclodextrin. In the presence of ethyl butyrate or glucose, butyric acid or gluconic acid (respectively) were produced as chemical messengers, triggering the opening of the pH-responsive molecular gate (via protonation of the benzimidazole units and rupture of the complex) to produce cargo release (a dye or drug) as the final output. Inspired by this work, other similar ensembles have been developed based on the rational combination of enzymatic effectors (as control unit) and molecular gates.^44−48^

**Figure 6:**
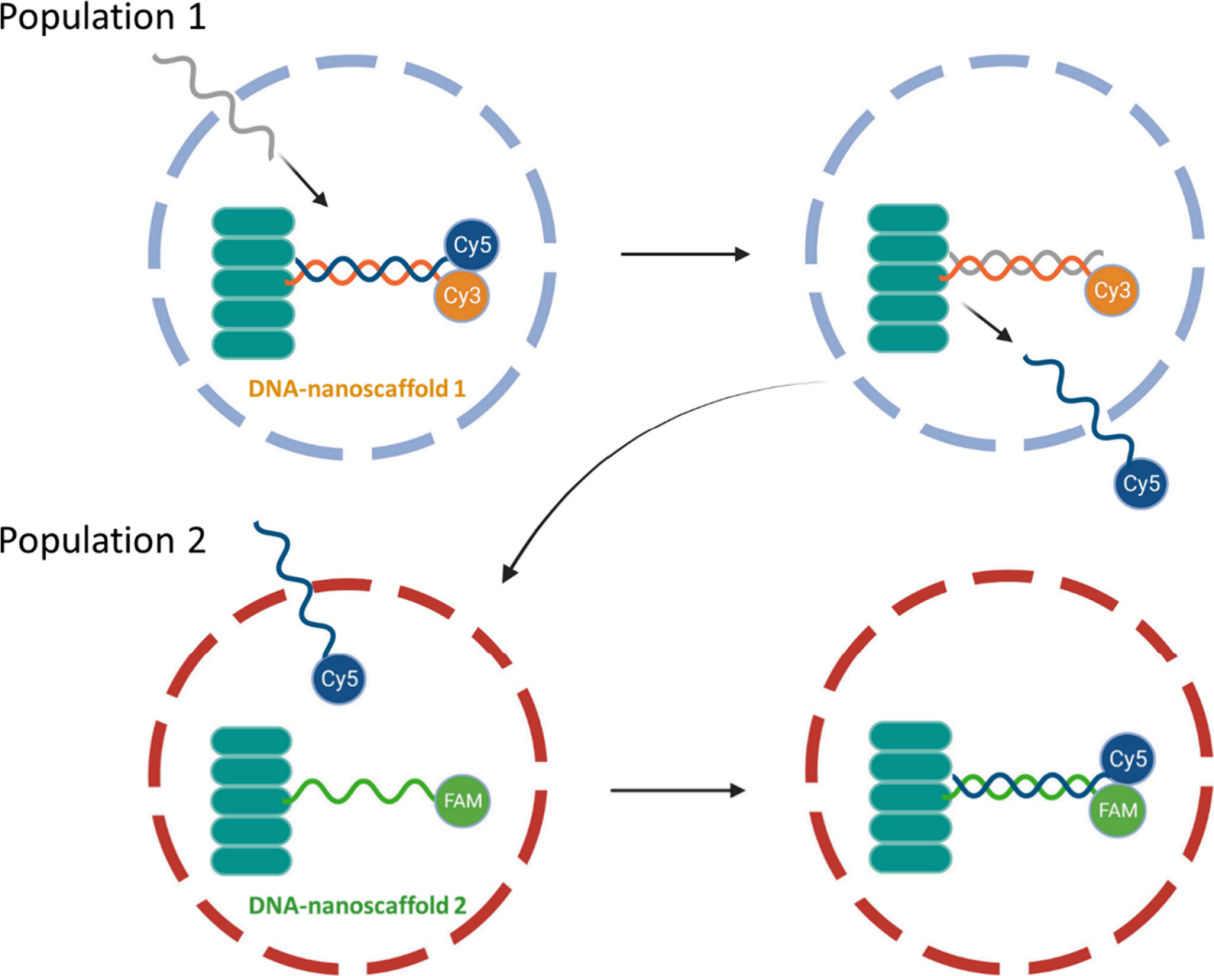
Coacervate to coacervate communication through DNA-displacement reactions, with a Cy5-marked DNA strand going from population 1 to population 2. Adapted with permission from ref (49). Copyright 2020 American Chemical Society.

**Figure 7:**
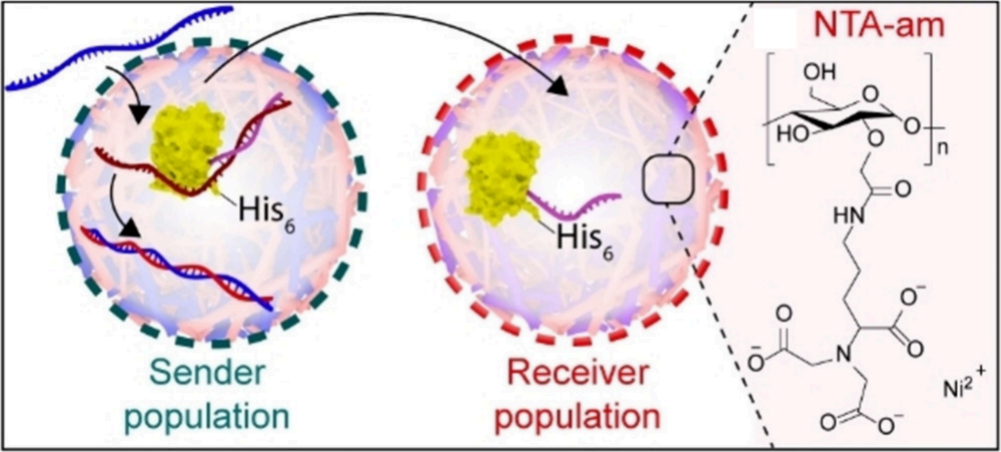
Coacervate to coacervate communication via DNA strands that allowed the exchange of His-tagged proteins. The protein-ssDNA released from senders is captured by receivers via His-Ni^2+^-NTA complexation. Reprinted with permission from ref (21). Copyright 2022 John Wiley and Sons. Distributed under a Creative Commons Attribution License 4.0 (CC BY).

Van Hest and co-workers developed a linear communication between two **coacervate** populations using **single stranded DNA sequences** as chemical messengers (Figure 6).^49^ In particular, supramolecular DNA-nanoscaffolds (self-assembled bispyridine-based stacks covalently decorated with DNA) were entrapped in coacervates and loaded with partially complementary (output) strands. Communication was triggered upon addition of a DNA input with a higher affinity toward DNA-nanoscaffold 1, inducing the release of the output strand. Then, output strand 1 (with a high affinity toward DNA-nanoscaffold 2) diffused as a chemical messenger to population 2. The same group also developed a **DNA-mediated** communication system that enabled transport of **proteins as chemical messengers** between sender and receiver coacervates (Figure 7).^21^ For this, proteins were attached to ssDNA via BCN-azide click chemistry and sequestered into coacervates by hybridization with a longer (uptake) ssDNA strand. A yellow fluorescent protein (YFP) containing His-tag moieties was employed for communication with a receiver population (containing Ni^2+^-NTA-amylose). Communication was triggered upon the addition of a releaser strand that displaced the YFP-ssDNA, which was then captured by the receiver population via the formation of His-Ni^2+^-NTA complexes. In this example, the use of fluorescent proteins enabled visualization by confocal fluorescence—yet the possibility to transport functional proteins remains a challenge for future studies.

**Figure 8:**
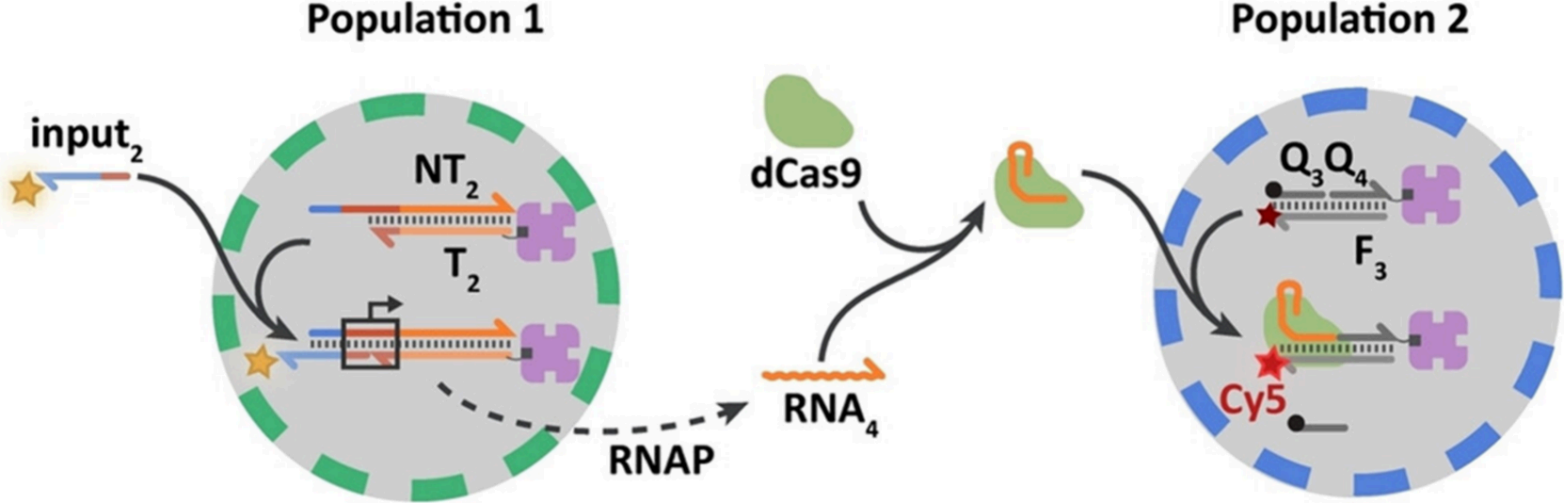
RNA-mediated communication between proteinosomes using CRISPR-Cas9 technology. An RNA sequence is transcribed and released from population 1 upon the addition of a specific DNA sequence. This RNA sequence, together with Cas9, guides the cleavage of a quenching DNA strand and fluorescence emission in population 2. Reprinted with permission from ref (50). Copyright 2022 John Wiley and Sons. Distributed under a Creative Commons Attribution License 4.0 (CC BY).

Also using **DNA** as messenger and **displacement reactions**, De Greef, Mann and co-workers pioneered communication between **proteinosomes**. In their work, the liquid interior of proteinosomes was loaded with streptavidin conjugated with biotinylated DNA strands, whereas the protein–polymer (BSA/PNIPAAm) membrane enabled the diffusion of short input/output strands to activate communication. Interestingly, the team also developed linear **light-activated** chemical communication using DNA strands with a photocleavable nitrobenzyl moiety. Upon irradiation with a confocal laser, the hybridized strand was cleaved into two shorter strands that dissociated from the sender proteinosome. Sender and receiver proteinosomes were spatially distributed in a microfluidic trapping array, which allowed for monitoring communication in space and time. The authors also developed a communication system that implemented CRISPR-Cas9 technology (Figure 8).^50^ In this case, senders transcribed and released RNA strands (upon input of DNA) as a messenger that guided the cleavage of the receiver’s population quenching strand by the action of endonuclease Cas9 (supplemented in the medium).

**Figure 9:**
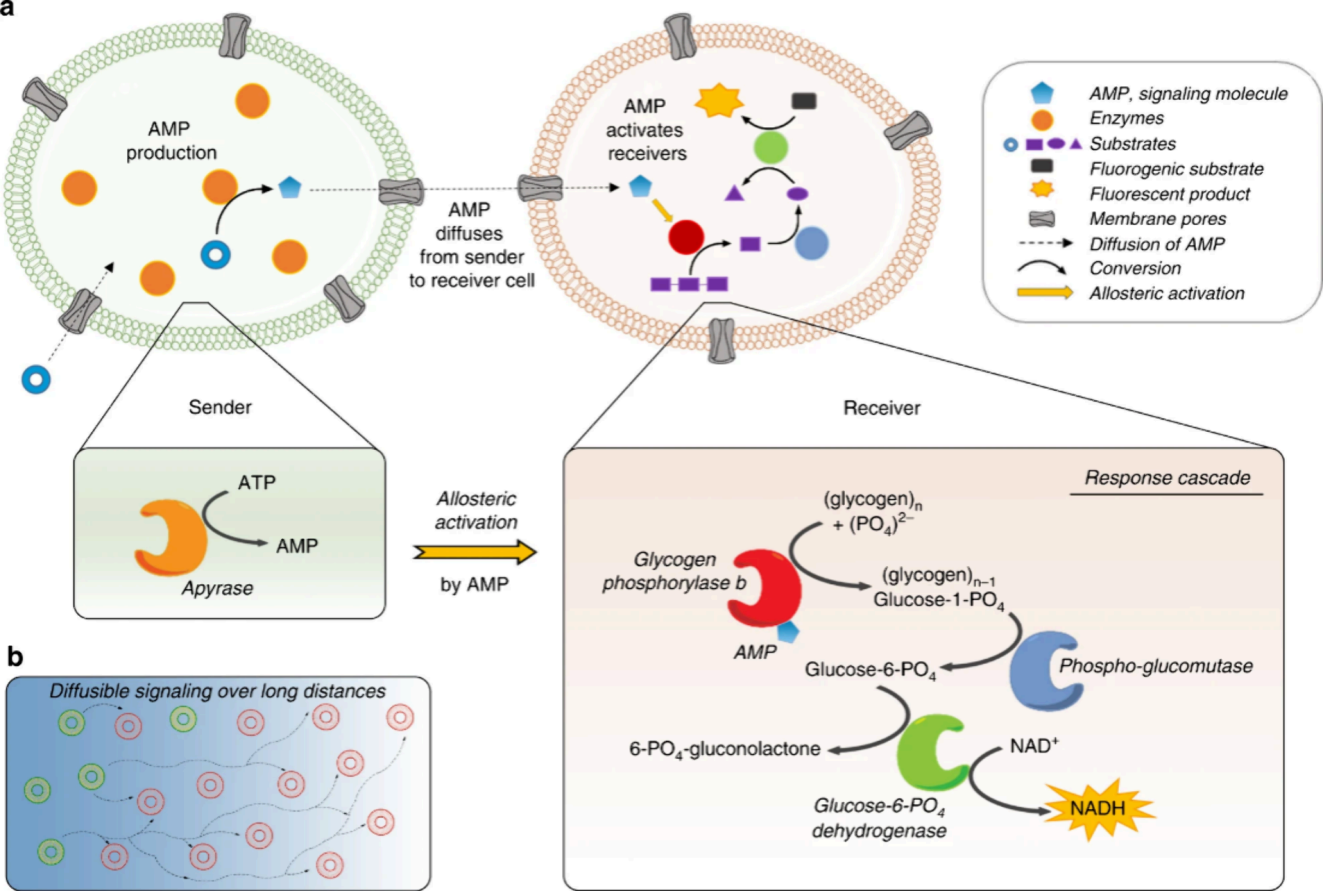
Communication between two populations of enzyme-loaded GUVs over long distances. The system is triggered by the addition of ATP, which is transformed into AMP by the sender population. AMP diffuses as a messenger to the receiver population, working as an allosteric activator which results in the production of NADH. Reprinted with permission from ref (3). Copyright 2020 Springer Nature. Distributed under a Creative Commons Attribution License 4.0 (CC BY).

One of the problems faced by communication is the dilution of chemical messengers, which can make the process unfeasible over certain distances. To deal with this issue, van Hest and co-workers designed two populations of **enzyme-loaded GUVs** able to communicate via allosteric amplification of a molecular signal (Figure 9).^3^ The sender GUV population contained the enzyme apyrase, whereas the receiver population contained glycogen phosphorylase b, phosphoglucomutase, and glucose-6-phosphate dehydrogenase. Both GUV populations were functionalized with α-hemolysin as a pore-forming protein to facilitate the passage of small substrates and products (while retaining enzymes in the GUV interior). By the external addition of ATP, sender GUVs generated AMP as a messenger for the receiver population. AMP acted as an allosteric activator for the enzymatic cascade entrapped in receiver GUVs, leading to the production of fluorescent NADH in the final step. Remarkably, the system could achieve efficient communication over distances as large as 200 times the particle diameter due to the allosteric activation mechanism.

**Figure 10:**
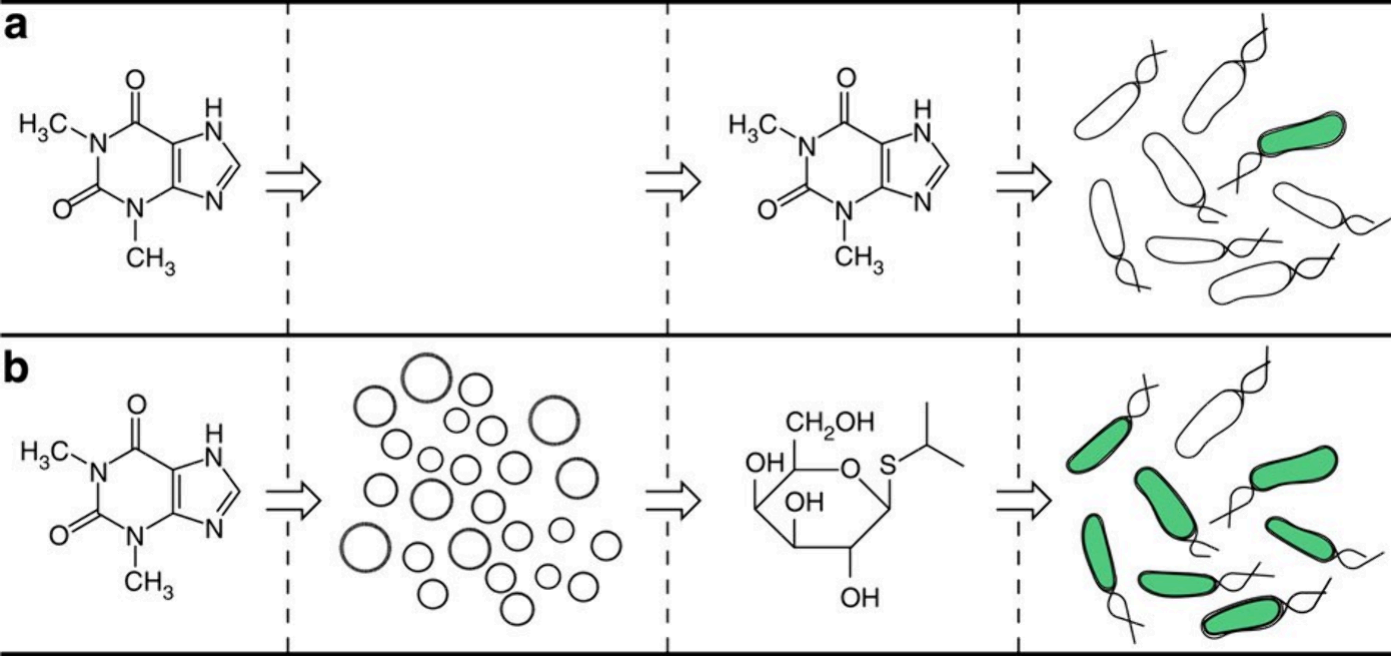
Linear communication between TXTL-loaded GUVs (artificial cells) and *E. coli* bacteria. (a) In the absence of artificial cells, bacteria do not respond to the presence of theophylline. (b) Artificial cells sense theophylline and release IPTG as a chemical messenger that activates GFP expression in bacteria. Reprinted with permission from ref (39). Copyright 2014 Springer Nature. Distributed under a Creative Commons Attribution License 4.0 (CC BY).

**Figure 11:**
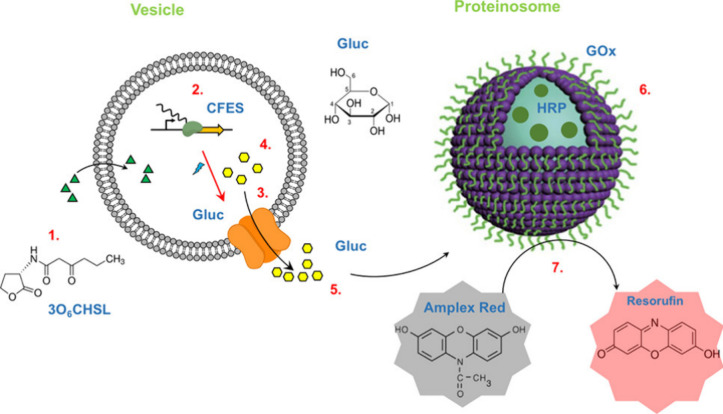
TXTL-based communication between GUVs and proteinosomes through the expression of a pore-forming protein inside GUVs, which is triggered by a quorum sensing molecule (3O6CHSL). After pore formation, glucose is released from GUVs and processed by proteinosomes to produce resorufin, a fluorescent dye. Reprinted with permission from ref (51). Copyright 2018 American Chemical Society.

**GUVs** loaded with **protein synthesis machinery** (TXTL) have been employed to design several communication systems. Although these systems have a limited stability and operation time (typically a few hours) due to the nature of TXTL extracts and consumption of chemicals, they have enabled GUVs to communicate with either other synthetic particles or living cells. In pioneering work in 2014, Mansy’s group established communication between TXTL-loaded GUVs (named as artificial cells) and *E. coli* bacteria, via the use of isopropyl β-d-1-thiogalactopyranoside (IPTG) as chemical messenger (Figure 10).^39^ Upon addition of membrane-permeable theophylline, GUVs expressed a pore-forming protein (α-hemolysin) that enabled the release of IPTG. This chemical messenger (IPTG) then induced *E. coli* to express green fluorescent protein as the output of the communication. In later work, Mansy and co-workers employed a similar approach to make GUVs communicate with enzyme-loaded proteinosomes (Figure 11).^51^ In this case, upon addition of a quorum sensing molecule *N*-(3-oxohexanoyl)-l-homoserine lactone (3O6CHSL), the expression of α-hemolysin induced the release of (membrane-impermeable) glucose as a chemical messenger. In response, proteinosomes processed glucose through a cascade reaction to produce fluorescent resorufin. In more recent work, Mansy’s group established communication between GUV-based artificial cells and eukaryotic cells (kidney or neural) via the release of brain-derived neurotrophic factor (BDNF) upon addition of external 3O6CHSL, producing protein expression or neural differentiation as a result.^52^ Other groups have employed TXTL machinery for constitutive (without external input addition)^53^ or light-controlled expression of different enzymes in GUVs, which subsequently catalyze the production of membrane-permeable gene inducers as chemical messengers to communicate with bacteria (that trigger the expression of fluorescent proteins as a response).^40^

### 3.2 Cascade Communication

**Figure 12:**
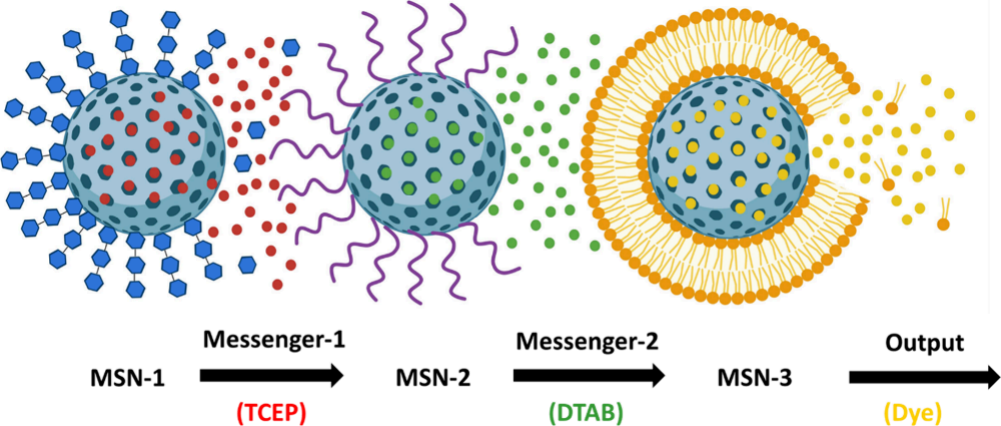
Schematic representation of cascade communication using MSNs, where the release of the first chemical messenger (red, TCEP) from MSN-1 triggers the molecular gate opening in MSN-2, based on the cleavage of dithiol-linked PEG chains. In consequence, a second messenger (green, DTAB) is released and perceived by MSN-3, disrupting the lipid bilayer surrounding this population, leading to dye release (yellow).

In pioneering work in 2014, Martínez-Máñez and co-workers developed a communication cascade between three different types of **gated MSNs** (Figure 12).^1^ MSN-1 was loaded with the reducing agent TCEP and capped with the oligosaccharide derivative Glucidex. MSN-2 was capped with PEG chains attached by disulfide bonds and loaded with the surfactant DTAB. Finally, MSN-3 was capped with a lipid bilayer and loaded with the dye Safranin O. The communication started in the presence of pancreatin, which induced the hydrolysis of Glucidex and release of TCEP from MSN-1. Subsequently, TCEP broke the PEG chains on MSN-2, triggering the release of DTAB as the second messenger. Finally, DTAB disrupted the lipid bilayer surrounding MSN-3, and the entrapped dye was released as the final output of the communication cascade. An important consideration when studying this type of communication system is to ensure that there is no interference between the different messengers and processes, which can be checked using incomplete nanoparticles.

Cascade communication has also been demonstrated by De Greef’s group using **DNA exchange between proteinosomes**—where the strand released by one population triggers the subsequent release of another strand in the next population—and by Bang-Ce Ye’s group using **DNA nanostructures on Au nanoparticles**.^24,25^

An interesting application of the cascade communication model is to connect different types of cells using micro/nanoparticles. In this regard, Mansy and co-workers were able to communicate between two different bacterial species using **artificial cells loaded with protein synthesis machinery**.^54^ In particular, the quorum sensing molecule (3O6CHSL) produced by *V. fischeri* bacteria activated the synthesis (and release) of *N*-(3-oxododecanoyl)-homoserine lactone (3OC12HSL) by artificial cells. In turn, 3OC12HSL activated a genetic construct in *E. coli* that resulted in the production of GFP.

**Figure 13:**
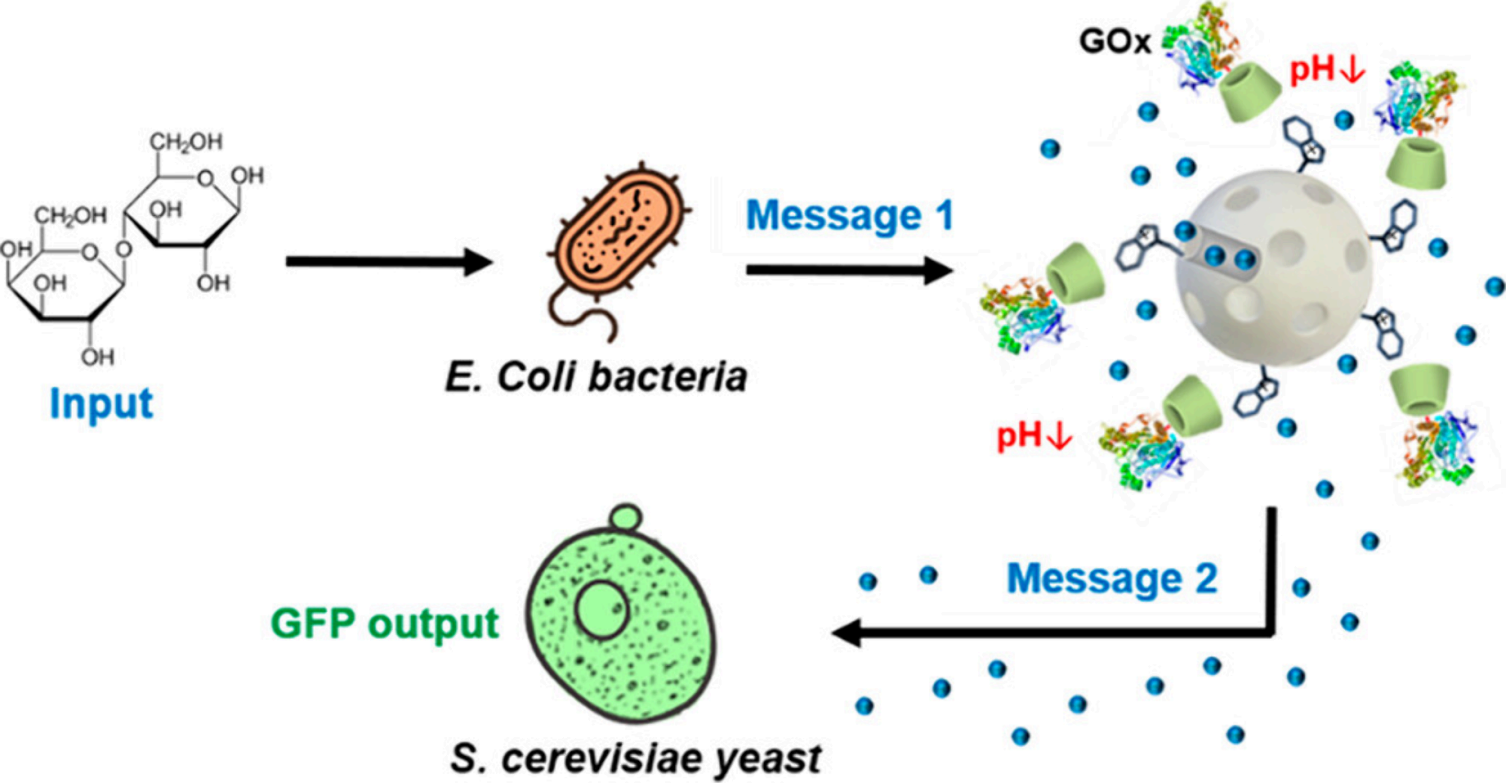
Schematic representation of a cross-kingdom communication system between *E. coli*, MSNs and *S. cerevisiae*, where engineered nanoparticles act as nanotranslators. After lactose addition, *E. coli* cells produce glucose as message 1, which is subsequently transformed by the GOx enzyme on MSNs, inducing the release of phleomycin as message 2 as a response. Finally, the presence of phleomycin induces GFP expression in *S. cerevisiae.* Reprinted with permission from ref (31). Copyright 2022 American Chemical Society.

In 2022, Martínez-Máñez’s group demonstrated **cross-kingdom communication** between two microorganisms, *E. coli* bacteria (prokaryotic kingdom) and *S. cerevisiae* yeasts (fungi kingdom), enabled by engineered **gated MSNs acting as nanotranslators**. MSNs were loaded with phleomycin, functionalized on the external surface with benzimidazole units and capped with glucose oxidase-modified cyclodextrin to form a pH-sensitive gate (Figure 13).^31^ Communication started upon input of lactose, which was hydrolyzed by β-galactosidase-expressing *E. coli* cells to produce glucose as a chemical messenger for the nanoparticles. Glucose was transformed into gluconic acid by glucose oxidase on the nanoparticle’s surface, which opened the pH-sensitive gate and induced the release of phleomycin (second chemical messenger). Finally, yeast cells sensed phleomycin and activated the expression of GFP. Furthermore, signal propagation was demonstrated with bacteria and yeast located on opposite ends in a microfluidic channel—showing that nanotranslator location in proximity of yeast cells resulted in more efficient communication compared to nanotranslator location in proximity of bacteria.

### 3.3 Interactive Communication

**Figure 14:**
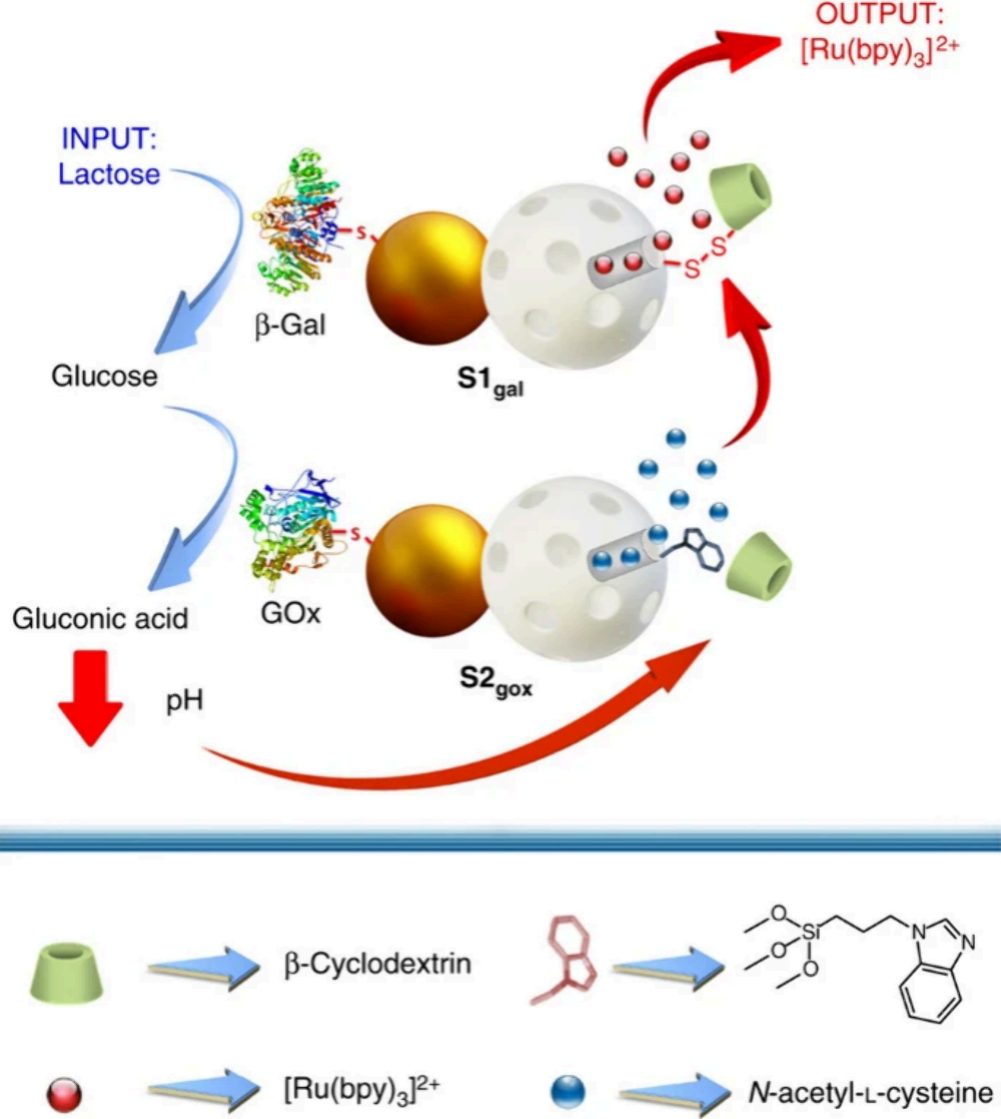
Interactive communication between two enzyme-functionalized Janus Au-MSN nanodevices (S1_gal_ and S2_gox_). The presence of lactose is perceived by S1_gal_ and hydrolyzed by β-Gal into glucose. S2_gox_ detects glucose and oxidizes it into gluconic acid by GOx activity, which induces *N*-acetyl-l-cysteine release from the MSN face. This reducing agent works as an interactive response from S2_gox_ to S1_gal_, cleaving the thiol-linked gate of S1_gal_ and inducing the release of [Ru(bpy)_3_]Cl_2_ as the final output. Reprinted with permission from ref (30). Copyright 2017 Springer Nature. Distributed under a Creative Commons Attribution License 4.0 (CC BY).

The engineering of interactive communication requires the design of micro/nanoparticles with a double sender/receiver role. The key here is to deploy and combine proper molecular machinery that is able to sequentially produce (e.g., enzymatically) and sense chemical messengers (e.g., using responsive ensembles such as molecular gates or DNA constructs). One of the first examples was reported by Martínez-Máñez and Villalonga’s groups. It involved communication between **Janus Au-MSNs** functionalized with enzymatic effectors and molecular gates, in which release from the first nanodevice occurred after receiving a response from a second nanodevice (Figure 14).^30^ Nanodevice-1 was loaded with a dye ([Ru(bpy)_3_]Cl_2_) and capped with disulfide-linked β-cyclodextrin on the MSN face, whereas the enzyme β-galactosidase was anchored to the Au surface. Nanodevice-2 was loaded with a reducing agent (*N*-acetyl-l-cysteine) and capped with a pH-responsive β-CD:benzimidazole nanovalve on the MSN face, whereas glucose oxidase was anchored on the Au surface. Communication started with the addition of lactose, which was hydrolyzed by β-galactosidase on nanodevice-1 into galactose and glucose. The glucose molecules diffused as a messenger to nanodevice-2, where glucose oxidase hydrolyzed it into gluconic acid. The resulting local drop in the pH led to the opening of the supramolecular gate and release of the entrapped *N*-acetyl-l-cysteine. This second molecular messenger acted as a signal for nanodevice-1, which finally resulted in the uncapping of the disulfide-linked gate and the release of the dye from nanodevice-1. In later work, the authors also developed a similar interactive communication system between nanoparticles triggered by addition of sucrose.^55^

**Figure 15:**
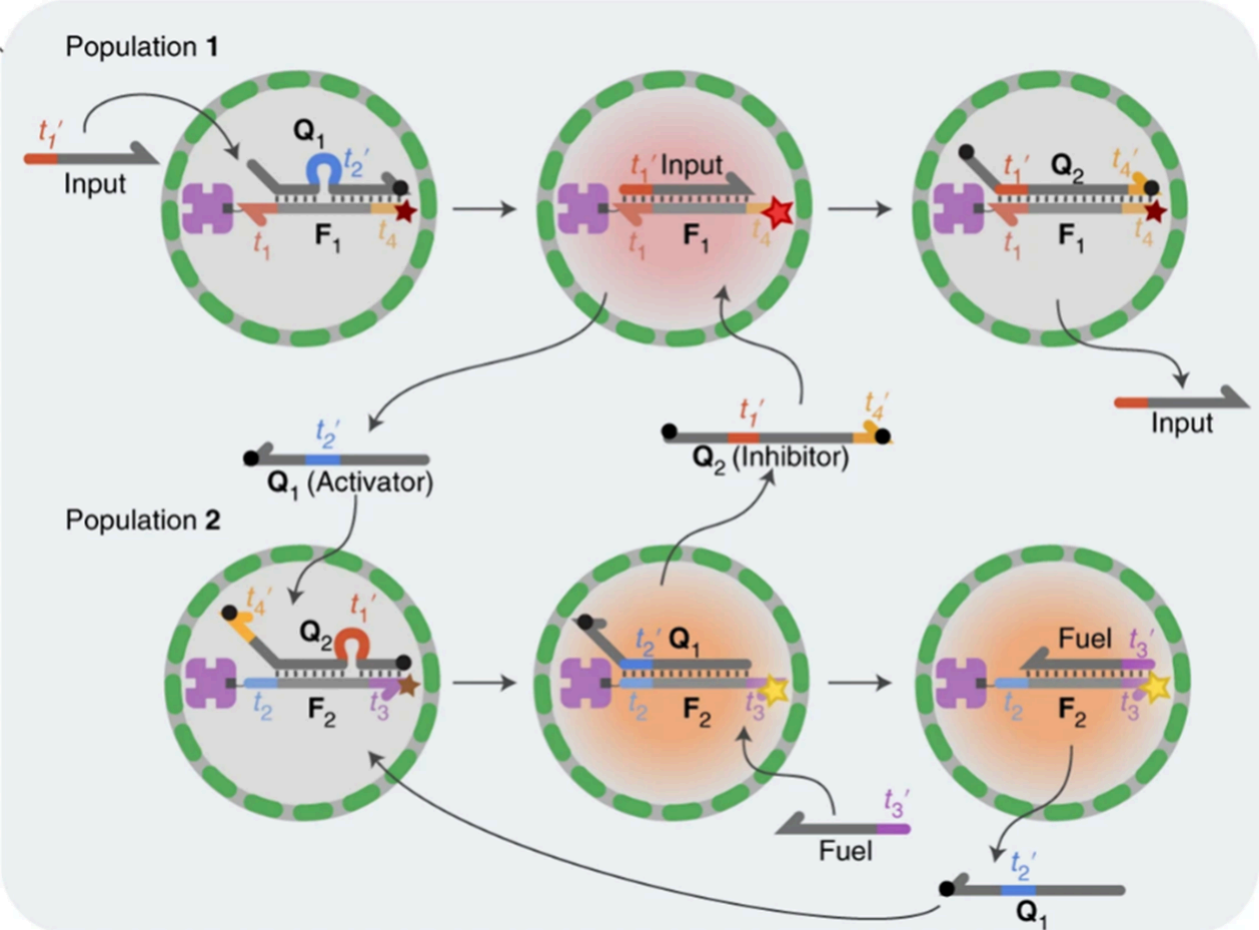
Interactive DNA-mediated communication between proteinosomes. Upon the addition of an input strand, population-1 activates fluorescence and releases a first messenger (activator) strand that diffuses inside population-2. Subsequently, population-2 releases a feedback (inhibitor) strand that goes back to population-1 and induces fluorescence quenching. Reprinted with permission from ref (24). Copyright 2019 Springer Nature.

Moreover, de Greef and co-workers developed interactive **DNA-mediated communication between proteinosomes** (Figure 15).^24^ The addition of a DNA input strand displaced a messenger (activator) strand from the first proteinosome (which turned on the fluorescence of this first population). The messenger (activator) strand was then captured by the second proteinosome population, inducing the release of a feedback (inhibitor) strand. The feedback (inhibitor) strand diffused back to the first population, inducing displacement of the input strand and fluorescence quenching. In addition, the process could be regulated by addition of a fuel strand, which displaced the activator strand from population-2 (thus, resulting in a higher availability of the activator strand and further signal propagation).

**Figure 16:**
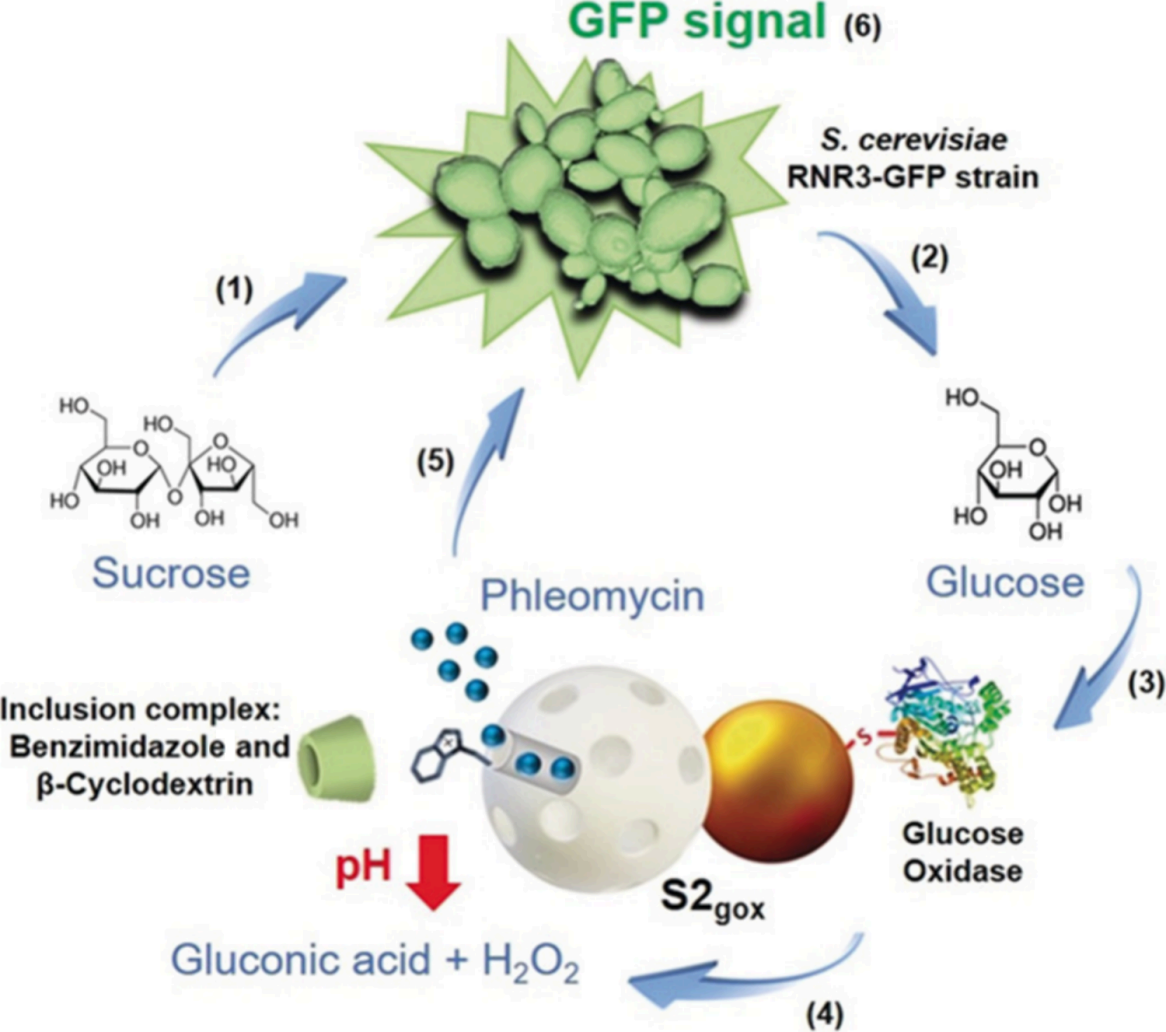
Interactive communication between yeast and nanoparticles. Upon input of sucrose, yeast cells produce glucose as a messenger for the nanoparticles. In turn, the nanoparticles sense glucose and release phleomycin as a second messenger that finally induces GFP expression in yeast cells. Reprinted with permission from ref (2). Copyright 2019 John Wiley and Sons.

In a step forward toward communication between nanoparticles and cells, Martínez-Máñez’s group developed a proof-of-concept interactive communication system between **Janus Au-MSNs and yeast cells** (Figure 16).^2^ The nanoparticles were loaded with phleomycin and functionalized with GOx on the Au face and a pH-responsive β-CD:benzimidazole nanovalve on the MSN face. Yeasts contained a genetic construct for GFP expression under the control of the RNR3 promoter, which activated in the presence of phleomycin. The communication started with the addition of sucrose as an input, which was converted by the invertase enzyme in yeast into glucose and fructose. Glucose acted as a chemical messenger for the nanoparticles, which triggered the release of phleomycin as a messenger for yeast cells. As a result, yeast cells became fluorescent due to GFP expression.

### 3.4 Circular Communication

**Figure 17:**
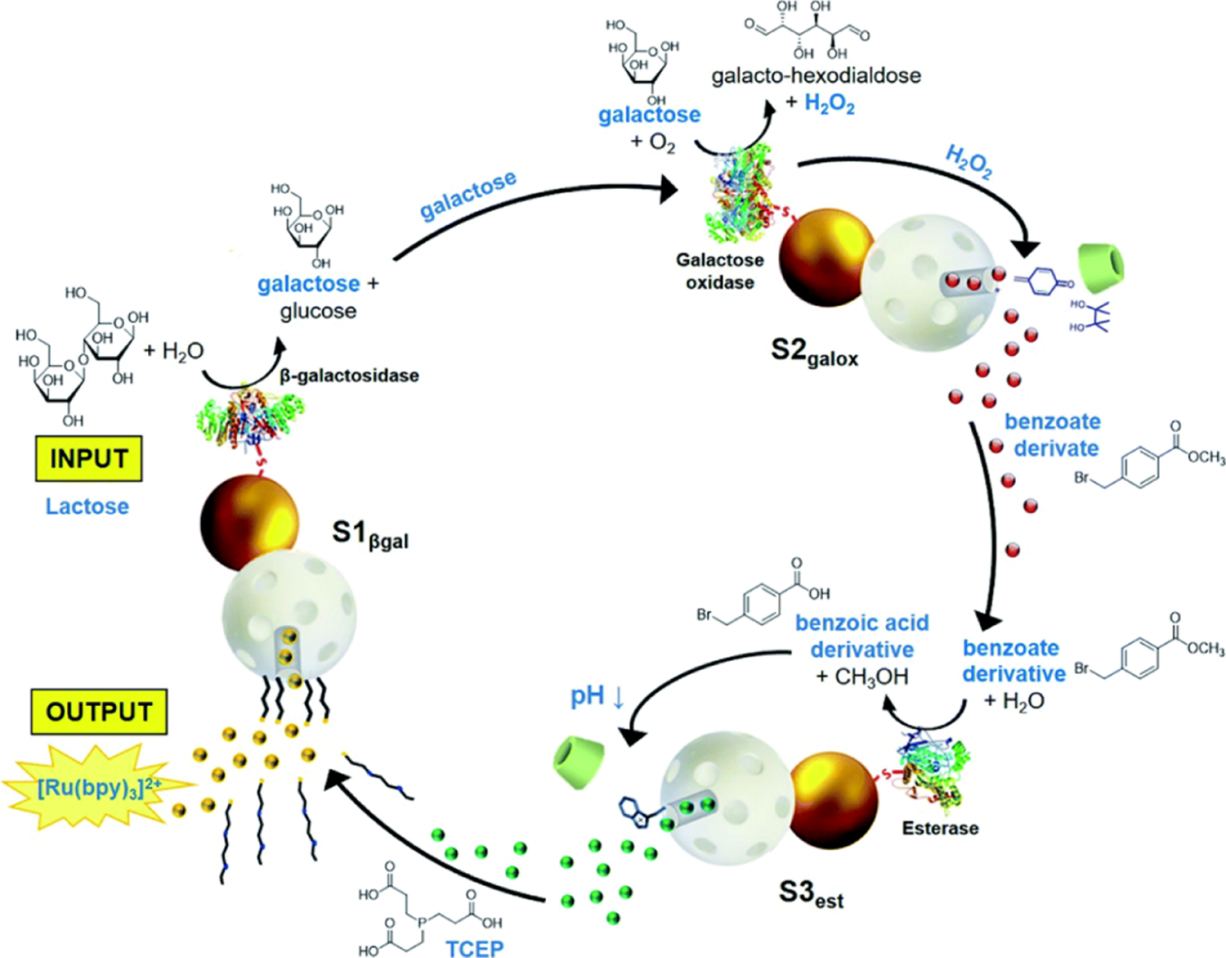
Circular communication between three Janus Au-MSNs populations. The system follows a sequence of enzymatic reactions and exchange of entrapped messengers between the nanodevices, which ends with the release of a dye from the same one that started the reaction chain. Reprinted with permission from ref (34). Copyright 2021 Royal Society of Chemistry.

Circular communication was achieved by Martínez-Máñez and co-workers by engineering three different enzyme-functionalized Janus Au-MSNs (Figure 17).^34^ Nanodevice-1 (S1_βgal_) was loaded with a dye ([Ru(bpy)_3_]Cl_2_) and functionalized with disulfide-linked PEG chains on the MSN face, whereas the enzyme β-galactosidase was anchored on the Au surface. Nanodevice-2 (S2_galox_) was loaded with an ester (benzoate) derivative and functionalized with a H_2_O_2_-sensitive gate on the MSN face, whereas the enzyme galactose oxidase was anchored on the Au surface. Finally, nanodevice-3 (S3_est_) was loaded with the reductive agent TCEP and functionalized with a pH-sensitive gate on the MSN face, whereas the enzyme esterase was anchored on the Au surface. The communication started upon the addition of lactose, which was transformed into galactose and glucose by β-galactosidase on nanodevice-1. Galactose was then transformed by galactose oxidase on nanodevice-2, resulting in the formation of H_2_O_2_ and subsequent release of the benzoate derivative. Next, this chemical messenger was transformed by esterase on nanodevice-3, resulting in the release of TCEP. Finally, the reductive cleavage of the disulfide-linked PEG chains on nanodevice-1 resulted in the emission of a fluorescent signal as the output of the communication. Interestingly, an increase in response time and decrease in output signal when increasing the number of communication steps was observed, which highlights the importance of analyzing these parameters in communication models.

**Figure 18:**
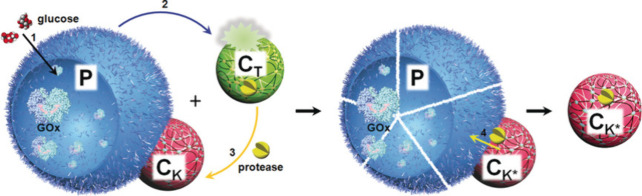
Circular communication between proteinosomes (P) and two coacervate populations (C_T_ and C_K_). Addition of glucose triggers the production of acid by P and the subsequent disassembly of C_T_ and sequestration of proteinase K by C_K_, finally resulting in P degradation by C_K_. Reprinted with permission from ref (56). Copyright 2019 John Wiley and Sons.

Another circular communication system using one proteinosome and two coacervate populations was developed by Mann’s group (Figure 18).^56^ It consisted of a population of glucose oxidase-loaded proteinosomes (P) attached to pH-resistant coacervates (C_K_), and a population of pH-sensitive proteinase K-loaded coacervates (C_T_). When in the presence of glucose, gluconic acid production by P induced a pH drop. The pH drop triggered the release of proteinase K from C_T_, which was then captured and concentrated in C_K_. The new proteinase activity acquired by C_K_ resulted in the degradation of the attached P as the output of the circular network. It is worth noticing that this approach (based on direct contact between different entities) mimics predatory behavior, yet their applicability is currently restricted to communication between artificial particles.

### 3.5 Stigmergy

**Figure 19:**
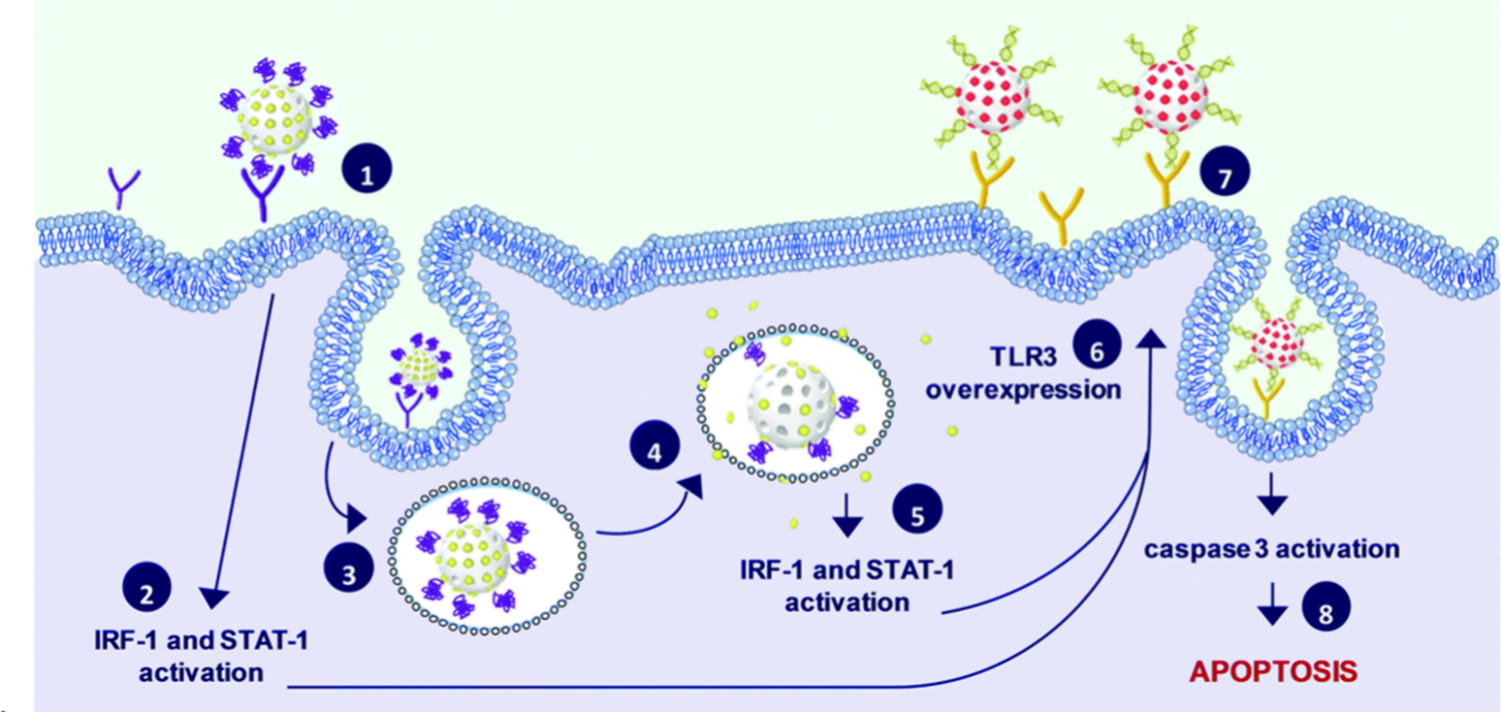
Schematic of communication between two different MSN populations by stigmergy in cancer cells. The internalization of the first population induces the overexpression of TLR3 receptors, thus facilitating the internalization of the second MSN population, which finally results in cell apoptosis. Reprinted with permission from ref (32). Copyright 2020 Royal Society of Chemistry.

Martínez-Máñez and co-workers have reported some pioneering work on nanoparticle communication by stigmergy, which holds great potential for biomedical applications. In this communication protocol, the trace left by an action in a medium of a first family of nanoparticles stimulates subsequent action by a second family of nanoparticles. In a first contribution, two different populations of MSNs were employed to induce apoptosis of cancer cells by stigmergy. The first population of MSNs was loaded with 9-*cis*-retinoic acid (RA) and capped with interferon-γ (IFN), whereas the second population was loaded with sulforhodamine B and capped with polyinosinic–polycytidylic acid (poly(I:C)), a synthetic agonist of TLR3 receptors (Figure 19).^32^ Thus, the first population of MSNs was internalized by cancer cells after IFN receptor recognition followed by intracellular release of the entrapped RA (due to degradation of the gating ensemble by lysosomal enzymes). Delivery of RA activated transcription factors that induced the overexpression of TLR3 receptors. Then, the second population of MSNs bound to TLR3 receptors via binding with poly(I:C), resulting in MSN internalization and activation of the caspase 3-dependent apoptotic pathway.

**Figure 20:**
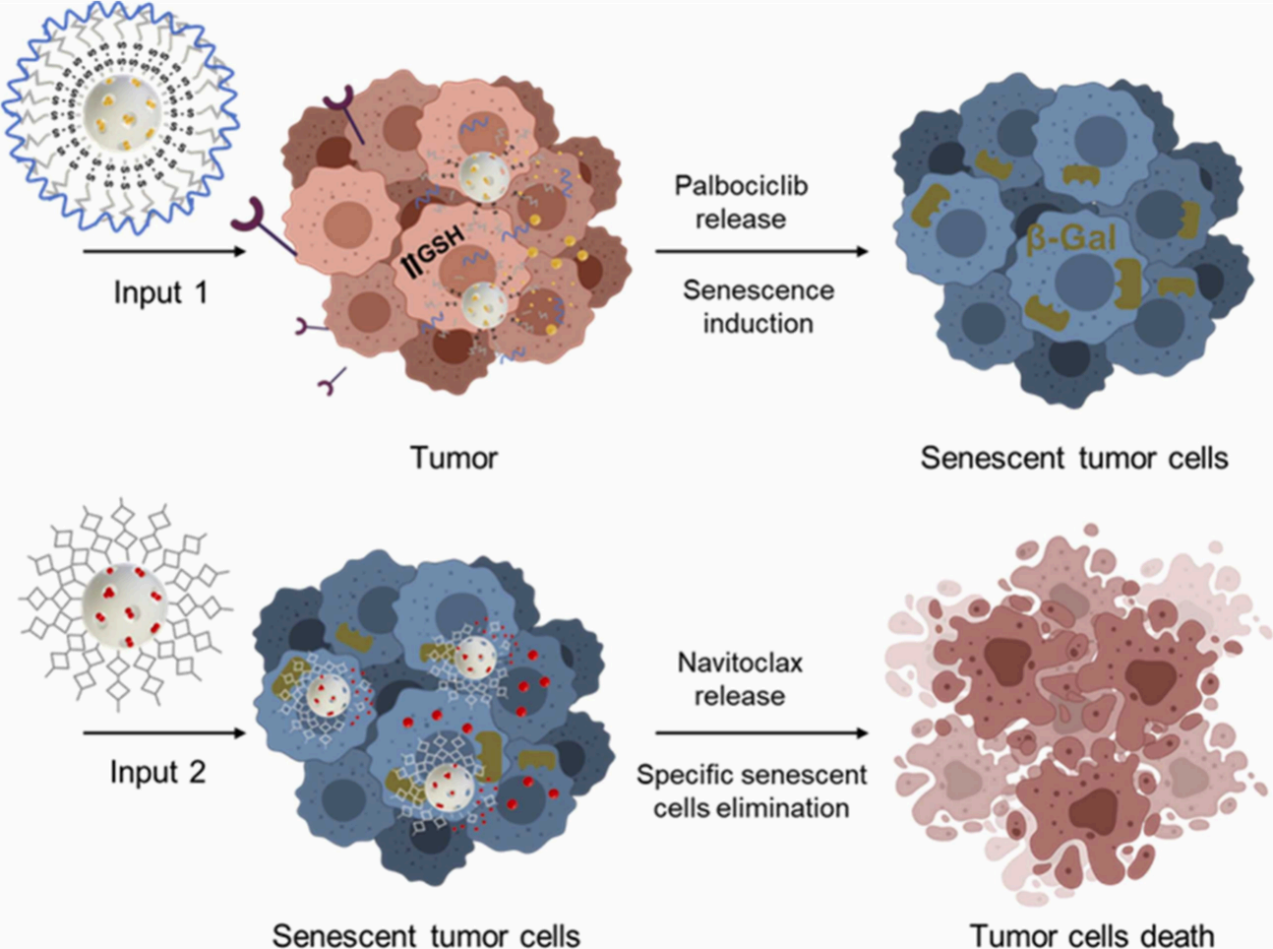
Communication between two populations of engineered MSNs by stigmergy, based on senescence induction in tumor cells. The first population of MSNs specifically interacts with cancer cells, releasing a senescent inductor. The second population of MSNs specifically targets senescent cells, inducing their lysis. Reprinted with permission from ref (4). Copyright 2023 Elsevier.

In more recent work, the same group leveraged nanoparticle–cell–nanoparticle communication to enhance cancer treatment *in vivo*, as demonstrated in a mouse model of human triple negative breast cancer (Figure 20).^4^ In this application, the first population of MSNs was loaded with the senescence-inducing drug palbociclib and capped with an MUC-1-targeting aptamer. The second population of MSNs was loaded with the senolytic navitoclax and coated with a hexa-oligosaccharide. The first population was able to target the MUC-1 receptor and induce senescence in breast cancer cells. Then, the second population was able to selectively eliminate tumor senescent cells. Remarkably, *in vivo* studies showed a reduction of metastasis and diminished side effects.

## 4 Conclusions and Prospects

This Account highlights advances in the development of different models of communication for micro/nanoparticles. Our efforts in this direction (and that of others) reflect two main approaches to design micro/nanoparticles with communication capabilities: the tailoring of nanoparticles with stimuli-responsive components and the engineering of micrometer-sized artificial cells. As discussed, the methodology for assembling a communicative micro- or nanoparticle starts with the selection of a proper particle chassis. Different contributions by our groups and others have shown the utility of MSNs, GUVs, coacervates, and proteinosomes; yet other types of particles such as polymersomes or even smart surfaces could be employed in future studies.^57^ The toolbox of technologies employed for information transmission and processing in these micro/nanoparticles embraces stimuli-responsive molecular gates, enzymes, DNA displacement reactions, and protein synthesis machinery (TXTL), which provides a wide range of possibilities for future studies. Yet, there is room for innovation in this area: other responsive molecular machinery could be designed and implemented, for instance, using synthetic chemistry (e.g., molecular motors),^58^ DNA nanotechnology, or protein engineering.

Based on how information flows between individual entities in the different reported networks presented throughout this Account, we propose a systematic classification into five main models of communication: linear, cascade, interactive, circular, and stigmergy. We believe this categorization will help in the design and modeling of communication networks. The different studies presented in this Account have shown the possibilities, at a technical level, of chemical communication between artificial particles and between artificial particles and biological cells. Interestingly, it has been demonstrated that engineering communication between particles and cells enables advanced functionalities such as establishing communication pathways between cells that would otherwise not interact or achieving enhanced therapeutic effects in the treatment of cancer. In this respect, communication by **stigmergy** is especially appealing as a new therapeutic approach for implementation in nanomedicine, based on the synergistic combination of different nanoparticles, to increase drug efficiency while minimizing side effects. The development of new stigmergy communication strategies may open new biomedical applications in the near future.

**Figure 21:**
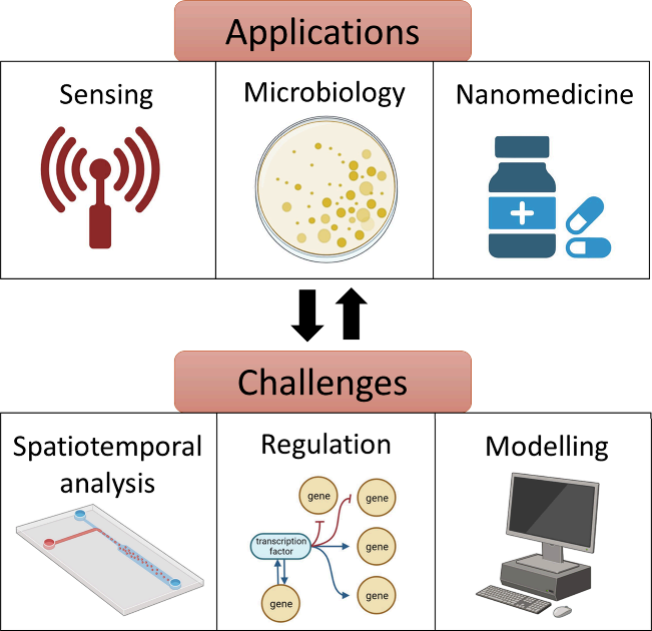
Schematic summary of applications and future challenges for chemical communication with micro/nanoparticles.

Nevertheless, most of the examples reported so far have focused on proof-of-concept demonstrations. We identify four main challenges to be addressed in future studies. First, it is important to raise the methodologies for quantitatively analyzing communication as a spatiotemporal phenomenon. In this regard, the use of microfluidic approaches and direct monitoring of communication processes (e.g., using real-time image acquisition with microscopy techniques) offer interesting prospects. Second, the reported examples still lack the level of complexity and regulation that occur in cellular communication. An approach to achieving regulation could be to employ enzyme inhibitors and activators as messengers in the communication process. Third, we foresee the need for collaboration between experimentalists and theoreticians to accomplish mathematical modeling and parametrization of communication processes.^59^ And finally, future studies should evolve toward the realization of practical applications in different areas (Figure 21). To name a few, some micro/nanoparticles able to communicate hold potential in the design of sensitive and selective sensing protocols with signal amplification features, in the regulation of bacterial communication and elimination, and in the development of new therapeutic approaches. Besides these examples, we believe that developing the tools for engineering and analyzing communication at the micro/nanoscale will lay the basis for a number of advanced biotechnological applications.
